# Neurofeedback for Pain Management: A Systematic Review

**DOI:** 10.3389/fnins.2020.00671

**Published:** 2020-07-16

**Authors:** Rubén Roy, Rocío de la Vega, Mark P. Jensen, Jordi Miró

**Affiliations:** ^1^Universitat Rovira i Virgili, Unit for the Study and Treatment of Pain–ALGOS, Department of Psychology, Research Center for Behavior Assessment (CRAMC), Tarragona, Spain; ^2^Center for Child Health, Behavior and Development, Children's Research Institute, Seattle, WA, United States; ^3^Department of Rehabilitation Medicine, University of Washington, Seattle, WA, United States

**Keywords:** systematic review, neurofeedback, neuromodulation, pain management, treatment outcome

## Abstract

**Background:** Chronic pain is a significant global health issue. For most individuals with chronic pain, biomedical treatments do not provide adequate relief. Given the evidence that neurophysiological abnormalities are associated with pain, it is reasonable to consider treatments that target these factors, such as neurofeedback (NF). The primary objectives of this review were to summarize the current state of knowledge regarding: (1) the different types of NF and NF protocols that have been evaluated for pain management; (2) the evidence supporting each NF type and protocol; (3) if targeted brain activity changes occur with NF training; and (4) if such brain activity change is associated with improvements on treatment outcomes.

**Methods:** Inclusion criteria were intentionally broad to encompass every empirical study using NF in relation to pain. We considered all kinds of NF, including both electroencephalogram- (EEG-) and functional magnetic resonance imagining- (fMRI-) based. We searched the following databases from inception through September 2019: Pubmed, Ovid, Embase, Web of Science, PsycINFO. The search strategy consisted of a combination of key terms referring to all NF types and pain conditions (e.g., neurofeedback, rt-fMRI-NF, BOLD, pain, migraine).

**Results:** A total of 6,552 citations were retrieved; 24 of these that were included in the review. Most of the studies were of moderate quality, included a control condition and but did not include a follow-up. They focused on studying pain intensity (83%), pain frequency, and other variables (fatigue, sleep, depression) in samples of adults (*n* = 7–71) with headaches, fibromyalgia and other pain conditions. Most studies (79%) used EEG-based NF. A wide variety of NF types and protocols have been used for pain management aiming to either increase, decrease or regulate brain activity in certain areas theoretically associated with pain.

**Conclusions:** Given the generally positive results in the studies reviewed, the findings indicate that NF procedures have the potential for reducing pain and improving other related outcomes in individuals with chronic pain. However, the current evidence does not provide definitive conclusions or allow for reliable recommendations on which protocols or methods of administration may be the most effective. These findings support the need for continued – but higher quality – research in this area.

## Introduction

### Rationale

Chronic pain is a major global health issue (Goldberg and Mcgee, 2011), affecting about one in four adults (Schopflocher et al., 2011; van Hecke et al., 2013; Nahin, 2015) and a similar number of youths (Huguet and Miró, 2008; King et al., 2011). Chronic pain has a number of negative physical, psychological and social consequences in the life for those with this condition (Institute of Medicine (U.S) Committee on a National Agenda for the Prevention of Disabilities, 1991; Bair et al., 2003; Finan et al., 2013; De Ruddere and Craig, 2016). The costs of chronic pain to society are enormous, and include both direct (e.g., medical expenses) as well as indirect costs [e.g., expenses associated with work absenteeism, hiring somebody to take care of the patients, or travel costs to receive treatment (Gaskin and Richard, 2012; Groenewald et al., 2014)]. For most individuals with chronic pain, the available treatments do not provide adequate relief and are generally unable to prevent new episodes (Williams et al., 2012).

The brain, an organ influenced by biological, psychological, and social factors, plays a central role in the onset and maintenance of pain (Chapin et al., 2012). For example, a growing body of evidence indicates that there are structural and functional neurophysiological brain abnormalities in individuals with chronic pain (May, 2008; Apkarian et al., 2011; Davis and Moayedi, 2013). Likewise, individuals with chronic pain evidence patterns of brain activity (as measured by electroencephalography; EEG) that differ from those without chronic pain (Pinheiro et al., 2016). It is possible that some of these brain abnormalities may be reversible with treatment (May, 2008; Flor, 2014). Thus, it would be reasonable to consider treatments that target brain activity directly as viable interventions for reducing the severity and impact of chronic pain.

Neurofeedback (NF) is a non-invasive treatment that targets brain activity. It is a type of biofeedback that provides real-time information to patients about their brain activity, allowing them to learn how to directly change this activity in ways that may lead to improved health and comfort. NF can be performed either by using brain activity measured via EEG or functional Magnetic Resonance Imaging (fMRI). The EEG approach is used much more often, because EEG biofeedback technology is more accessible and less expensive. With EEG-based NF, one or more electrodes are placed on the patient's scalp to measure the amplitude (also referred to as “power”) of oscillatory activity in different frequency bandwidths. The raw electrical signal represents the collective activity of millions of neurons in the cortex, just below the electrode. This signal is analyzed and aspects of that electrical brain activity are fed back to the patient (Jensen et al., 2014). Normally, EEG-based NF targets a change in the power of activity in specific oscillation bandwidths whereas fMRI-based NF targets changes in the blood oxygen-level dependent (BOLD) activity in regions of interest in the brain (Sulzer et al., 2013; Thibault et al., 2018).

Whether NF is conducted with EEG or fMRI, measured changes in brain activity are fed back to the patient. Often, but not always, the feedback is provided via a game. For example, a program might allow the patient to “fly” a plane when he or she makes a change in the targeted brain activity (e.g., an increase in alpha power as measured over the sensory cortex). The plane will fly smoothly as long as the targeted brain activity is in the direction of the training criteria established by the therapist, whereas the plane might drop or otherwise malfunction if the brain activity falls outside of the training range. This feedback influences and progressively helps the patient learn to change brain activity via operant conditioning (Heinrich et al., 2007; Sherlin et al., 2011). It is important to note that although operant conditioning is the principle underlying the most common NF treatments, there are some types of NF that operate via different principles (Sherlin et al., 2011). Also, changes in brain activity often take a relatively long time to occur with NF treatment; a full course of NF treatment is normally comprised of 15–50 sessions of 20–40 min each (Heinrich et al., 2007; Hammond, 2011).

In the context of pain treatment, NF aims to change brain activity that is thought to underlie or influence the experience of pain (Ibric and Dragomirescu, 2009). The findings from a number of research studies provide preliminary support for the efficacy of NF for reducing pain in clinical samples (Jensen et al., 2014; Miró et al., 2016). However, some investigators have questioned whether NF has any beneficial effect for pain or other problems over and above placebo or outcome expectancy effects (Thibault et al., 2017). Thus, a critical summary of the available evidence regarding the efficacy of NF interventions targeting pain as an outcome is needed in order to better understand the current state of knowledge regarding this potentially promising pain intervention.

### Objectives

Given the considerations discussed above, the primary objectives of this review were to summarize the current state of knowledge regarding (1) the efficacy of NF for reducing pain and (2) the effects of NF on pain-related brain activity in individuals experiencing pain.

### Research Questions

Specifically, we aimed to: (1) describe the different types of NF and NF protocols, and how NF has been used for pain management; (2) summarize the evidence regarding the efficacy of each type of NF and different NF protocols for modulating pain and for improving pain-related outcomes; (3) determine the level of evidence regarding the effect of NF training on measures of brain activity thought to be related to pain, and if changes in measures of this brain activity are associated with improvements in pain-related outcomes; and (4) asses the quality of the studies included in the review.

## Methods

### Study Design

The current systematic review was conducted and reported following the Preferred Reporting Items for Systematic reviews and Meta-Analyses for Protocols 2015 (PRISMA-P 2015) guidelines (Moher, 2015) and was preregistered at the PROSPERO International Prospective Register of Systematic Reviews (https://www.crd.york.ac.uk/prospero/; with registration number CRD42018115335).

### Participants, Interventions, Comparators

We included studies using samples of children or adults, either healthy or with clinical pain conditions, where neurofeedback was used to influence pain outcomes. The inclusion criteria were intentionally broad in order to include in the review every empirical study using NF to treat pain. All types of studies were included, regardless of sample size or study design. We also considered all kinds of NF, both EEG- and fMRI-based NF, and included studies combining the use of NF with other interventions or using NF to enhance the efficacy of other pain treatments. We also aimed to include studies on all types of pain, including chronic pain, acute pain, and laboratory (induced) pain. Any study that assessed at least pain intensity or pain frequency was included. The only exclusion criterion was if a given paper under consideration was written in a language other than Spanish or English.

We considered studies that included the assessment of pre- to post- treatment changes in pain intensity and/or pain frequency, as measured using questionnaires or rating scales with support for their reliability and validity (Jensen and Karoly, 2001). When available, we also examined the extent to which any changes noted after NF training did or did not maintain at follow-up.

When assessed, we noted the effects of NF on pain-related outcomes, including fatigue, sleep problems/sleep quality, psychological function (anxiety or depression), perceived health-related quality of life and pain-related interference or disability. We also considered pre- to post-treatment changes in measures of brain activity; that is, pre- to post-treatment changes in the power of different brain oscillation bandwidths or pre- to post-treatment changes in BOLD activity. When possible, we also examined if any pre- to post-treatment improvements in these outcomes maintained at follow-up.

### Search Strategy

We searched the following databases from inception through September 2019: PubMed, Ovid, Embase, Web of Science, PsycINFO and Scopus. The search strategy consisted of a combination of key terms referring to all neurofeedback types and pain conditions (e.g., neurofeedback, rt-fMRI-NF, pain, migraine, fibromyalgia). To see the full Pubmed strategy please see https://www.crd.york.ac.uk/PROSPEROFILES/115335_STRATEGY_20181031.pdf. We also searched the reference lists of all articles reviewed in order to identify any additional studies to include. In addition, we performed a search of ClinicalTrials.gov to identify ongoing or completed studies with unpublished results and asked the corresponding authors to allow us to include their results in the review. Finally, we attempted to contact the authors of any papers included in the review that did not provide all the data needed for our synthesis to request these data.

### Data Sources, Studies Sections, and Data Extraction

Two of the authors (RR and RdlV) independently assessed the eligibility of the articles retrieved after the database search for inclusion in the review. If any disagreement emerged, they were resolved in consultation with a third author (JM). Next, a deduplication process was conducted via a reference manager (Mendeley). Once a final list of selected articles was identified, their reference lists were reviewed to identify additional studies that could be of interest.

We extracted the following study characteristics from each article identified for inclusion: article title, author(s), publication year, country, sample characteristics (sample size, age, sex, education level, household income, pain problem), intervention protocols (i.e., scalp positions and bandwidths targeted for EEG-based NF, brain regions being targeted in fMRI-based NF, number, duration and frequency of sessions), primary study outcomes (i.e., pain intensity, pain frequency), and secondary outcomes (i.e., fatigue, sleep quality, psychological function [anxiety, depression], perceived health-related quality of life and pain-related disability). If available, we extracted EEG or BOLD activity in whichever way it was reported.

When more than one measure was used to assess the same construct, we planned to inform about the one that is reported most often in the literature as the primary outcome for that study. If data from the same study were reported in different papers, we only retrieved the data from the paper that was published first, unless there was a subsequent study that added additional participants or provided additional data.

### Data Analysis

Given the paucity of research on the topic, as evidenced by preliminary searches as well as the disparity of methods and outcomes reported, we anticipated that a meta-analytical approach would not be feasible. As this was confirmed after the search, here we present a systematic narrative synthesis summarizing the characteristics and findings of the studies included in the review. We included all studies identified irrespective of their risk of bias. In addition, we organized the narrative synthesis by study design, starting with those with stronger designs and continuing from there to the studies using lower-quality designs. We describe separately EEG-based NF (and its subtypes) and fMRI-based NF. We report on the outcomes (clinical and neurophysiological) as a function of the type of NF (EEG- or fMRI-based) and protocol used. We also summarize the different uses of NF in pain management. Next, we summarize NF's effects on pain intensity and pain frequency, as well as on measures of the pain-related variables mentioned above. We also note whether the studies provided EEG- or fMRI-assessed physiological data, and if they reported changes in measures of physiological activity following NF. If so, we assessed whether these changes in brain activity were associated with changes in the brain activity targeted by the intervention. If presented by the study authors, we also report on the extent to which changes in measured brain activity change were associated with observed improvements in treatment outcomes.

In addition, we rated and describe study quality using the Quality Assessment Tool for Quantitative Studies from the Effective Public Health Practice Project [EPHPP; (Thomas et al., 2004)], as this tool allows for a comparison of study quality between studies using different designs. The EPHPP tool consists of six quality components to be rated as “strong” (coded as “1”) “moderate” (coded as “2”), or “weak” (coded as “3”): selection bias, study design, confounders, blinding, data collection methods, and withdrawals and drop-outs. We did not compute a final score for each study as relevant methodological aspects of the studies appear to be better assessed individually (Jüni et al., 1999). Again, two authors (RR and RdlV) conducted this evaluation independently. In the event of any disagreements, these were resolved in consultation with a third author (JM).

## Results

### Study Selection and Characteristics

**Figure 1:**
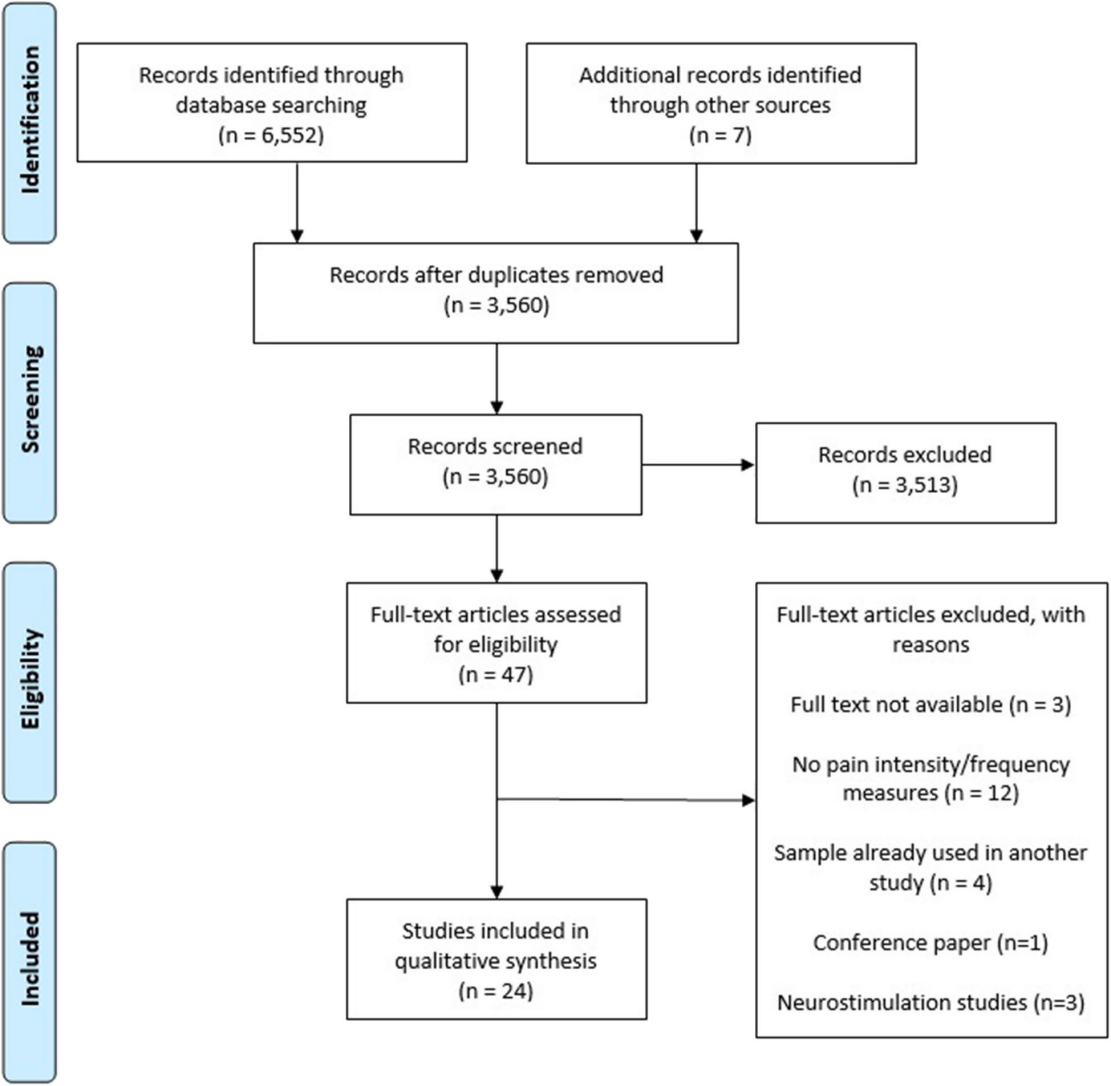
Study selection diagram flow.

Our initial search retrieved 6,552 citations. After eliminating duplicates, 3,560 articles were assessed based on their title and abstract. A total of 3,513 articles were excluded because they did not meet the inclusion criteria and 47 were read in full. A total of 11 authors were contacted for additional data. However, only one of these responded to us, and this author did not provide the additional data needed. One completed project that could be potentially eligible was found in ClinicalTrials.gov. We contacted the corresponding author for that project but did not receive an answer. The final number of studies included in the review was 24. See Figure 1 for a flow diagram of the article selection process.

The vast majority of the studies we identified for inclusion in this review were conducted in the last decade. A plurality of the studies (*k* = 12, 50%) were conducted in the United States, four (17%) were conducted in Germany, and the rest were conducted in six other countries. The quality of the study designs was rated as “moderate” for the most part. Two studies (9%) were case series, 19 (79%) were non-randomized trials, and only three (13%) were randomized controlled trials (RCTs). The sample sizes in the studies that were not case series ranged from *n* = 7–71. Only seven (29%) studies included follow-up assessments. Most of the studies (19, 79%) included only adults, four (17%) included both adults and youths, and one (4%) used a pediatric sample only. The pain type most frequently studied was headache (including migraines; *k* = 5, 21%). The rest of the studies evaluated the effects of NF in individuals with a variety of pain conditions: fibromyalgia (two studies), spinal cord injury (SCI) and chronic pain (three studies), a variety of chronic pain problems (two studies), pain associated with radiation therapy for cancer (one study), chemotherapy-induced peripheral neuropathy (CIPN; one study), postherpetic neuralgia (one study), Complex Regional Pain Syndrome Type I (CRPS-I; one study), and chronic paraplegia (one study). Two studies (8%) used NF to enhance hypnotic analgesia in individuals with multiple sclerosis. Also, a total of four studies (17%) assessed the effects of NF on laboratory (induced) pain in healthy individuals.

In addition to pain intensity (*k* = 20, 83%) and pain frequency (*k* = 4, 17%), the studies assessed a number of other pain-related outcomes such as: fatigue (*k* = 6, 25%), sleep quality/problems (*k* = 3, 13%), anxiety (*k* = 2, 8%), depression (*k* = 2, 8%), and pain-related interference (*k* = 4, 17%). Seventeen (71%) of the studies assessed changes in brain activity after the intervention. Of these, 11 (46%) performed analyses to determine if pre- to post-treatment changes in measures of brain activity were associated with pre- to post-treatment changes in one or more study outcomes.

Regarding the NF type, most studies (*k* = 19; 79%) used EEG-based NF; five (*k* = 5, 21%) used fMRI-based NF. Among the studies that were conducted with EEG, 15 (63%) used brain oscillation power-based NF, two (8%) used surface and/or low-resolution electromagnetic tomography (LORETA) Z-score NF, and two (8%) used event related potentials (ERPs) NF. A total of 21 studies (88%) used NF as a single intervention, one (4%) used it in addition to other interventions and two (8%) used it to enhance the effects of another intervention.

A variety of control conditions were used in the controlled studies: one study (4%) tested NF provided to a clinical sample against the same NF intervention provided to a control sample of healthy individuals and a waitlist-control condition, one (4%) used an active control condition and a waitlist-control condition, two (8%) used a waitlist-control condition, one (4%) used a sham condition, four studies (17%) used an active control condition, one (4%) used three active control conditions and a sham condition, and one (4%) used four sham control groups and one active control condition.

Participants in the studies reviewed received between one to 98 sessions. For those who received more than one session, frequency ranged from once a week to daily, and duration ranged from 16–120 min. See Tables 1, 2 for details about the interventions and participants in the studies reviewed.

**Table 1:** Description of participant characteristics.

**Authors (year)**	**Condition**	**Sample size**	**Age M (SD or Range)**	**Sex (% female)**	**Sample condition**
Caro and Winter (2011)	E: NF	15	66.7 (12.3)	93	Fibromyalgia
	C: TAU	63	50.5 (13.9)	79	
DeCharms et al. (2005)	E: NF or biofeedback	12	36.7 (31–38)	33	Chronic pain
	C: Healthy control group	36	23.5 (18–37)	44	Healthy sample
Emmert et al. (2014)	E: NF (lAIC)	14	27.6 (2.1)	50	Healthy sample
	E: NF (ACC)	14	27.4 (2.6)	50	
Farahani et al. (2014)	E: NF	15	37.6 (7.5)	47	Headache
	E: TENS	15	40.7 (10.1)	40	
	C: WL	15	37.3 (9.4)	47	
Guan et al. (2015)	E: NF	8	58.5 (2.4)	37	Postherpetic neuralgia
	C: Sham NF	6	61.3 (3.4)	50	
Hasan et al. (2015)	E: NF	7	50 (4)	14	Central neuropathic pain and chronic paraplegia
Jacobs and Jensen (2015)	E: NF	4	NR (14–56)	50	Variety of chronic pain problems
Jensen et al. (2018)	E: NF + Hypnosis	12	57.5 (10.6)	75	Multiple sclerosis with either chronic pain, fatigue or both
	E: Mindfulness + Hypnosis	10			
	C: Hypnosis	10			
Jensen et al. (2013a)	E: NF	10	46.1 (12.6)	30	Spinal cord injury and chronic pain
Jensen et al. (2016)	E: NF + Hypnosis	10	49.2 (11.26)	63	Multiple sclerosis and chronic pain
	E: Relaxation + Hypnosis	9			
Jensen et al. (2007)	E: NF	18	40.8 (17–56)	89	CRPS-I
Jensen et al. (2013b)*	E: NF	30	49.2 (22–77)	27	Spinal cord injury and chronic pain
	E: tDCS	28			
	E: Hypnosis	29			
	E: Concentration meditation	30			
	C: Sham tDCS	30			
Kayiran et al. (2010)	E: NF	18	31.8 (6.2)	100	Fibromyalgia
	C: Escitalopram	18	32.4 (6.7)	100	
Koberda et al. (2013)	E: NF	4	NR (46–59)	50	Variety of chronic pain problems
Mathew et al. (1987)	E: NF	8	NR (18–40)	NR	Tension headache
	C: WL	4			
Miltner et al. (1988)	E: NF	10	NR (21–46)	0	Healthy sample
Prinsloo et al. (2019)	E: NF	14	56 (35–76)	21	Patients with head and neck cancer undergoing radiation therapy
Prinsloo et al. (2018)	E: NF	35	62 (9.6)	89	Chemotherapy-induced peripheral neuropathy
	C: WL	36	63 (11)	86	
Rance et al. (2014a)	E: NF	10	27.8 (4.7)	60	Healthy sample
Rance et al. (2014b)	E: NF	10	29 (6.4)	40	Healthy sample
Siniatchkin et al. (2000)	E: NF	10	10.5 (1.5)	20	Migraine
	C: Healthy control group	10	9.9 (0.6)	30	Healthy sample
	C: WL	10	11.6 (2.6)	20	Migraine
Stokes and Lappin (2010)	E: NF	37	NR (9–79)	78	Migraine
Vučković et al. (2019)	E: NF	15	50.6 (14.1)	20	Central neuropathic pain and chronic spinal cord injury
Walker (2011)	E: NF	46	NR (17–62)	NR	Migraine

NR, not reported; E, Experimental; C, Control; NF, neurofeedback; TAU, Treatment as usual; WL, Wait-list control group; TENS, Transcutaneous electrical nerve stimulation; CRPS-I, Complex regional pain syndrome type I; TBI, Traumatic brain injury; CIPN, Chemotherapy-induced peripheral neuropathy. lAIC, Left anterior insular cortex; ACC, Anterior cingulate cortex; tDCS, transcranial Direct Current Stimulation. ^*^In this study the same participants received up to a single session of all four active procedures and the sham control procedure.

**Table 2:** Description of study and intervention characteristics.

**Authors (year)**	**Country**	**Study design**	**NF type**	**Monotherapy (Yes / No)**	**Number of sessions**	**Length of sessions (minutes)**	**Follow-up**
Caro and Winter (2011)	USA	Cohort analytic	Frequency NF	Y	Varied *M* = 58 range (40–98)	NR	None
DeCharms et al. (2005)	USA	Cohort analytic	rt-fMRI NF	Y	1	13 to 39	None
Emmert et al. (2014)	Switzerland	Cohort	rt-fMRI NF	Y	1	16	None
Farahani et al. (2014)	Iran	RCT	Frequency NF	Y	15	30	None
Guan et al. (2015)	China	Cohort analytic	rt-fMRI NF	Y	1	NR	None
Hasan et al. (2015)	UK	Cohort	Frequency NF	Y	Varied range (2–40)	45	1 month
Jacobs and Jensen (2015)	USA	Case series	Frequency NF	Y	Varied range (22–41)	30	None
Jensen et al. (2018)	USA	Cohort analytic	Frequency NF	N	6	30	1 month
Jensen et al. (2013a)	USA	Cohort	Frequency NF	Y	12	NR	Varied (3 months)
Jensen et al. (2016)	USA	Cohort analytic	Frequency NF	N	4	30	1 month
Jensen et al. (2007)	USA	Cohort	Frequency NF	N	1	30	None
Jensen et al. (2013b)	USA	Cohort analytic	Frequency NF	Y	1	20	None
Kayiran et al. (2010)	Turkey	RCT	Frequency NF	Y	20	30	None
Koberda et al. (2013)	USA	Case series	Surface Z-score and LORETA NF	Y	Varied range (10–65)	30	None
Mathew et al. (1987)	India	Cohort analytic	Frequency NF	Y	20	30	None
Miltner et al. (1988)	Germany	Cohort	ERP-based NF	Y	1	120	None
Prinsloo et al. (2019)	USA	Cohort	Z-score LORETA NF	Y	Varied range (1–6)	20	None
Prinsloo et al. (2018)	USA	RCT	Frequency NF	Y	20	45	1 month 4 months
Rance et al. (2014a)	Germany	Cohort	rt-fMRI NF	Y	4	40	None
Rance et al. (2014b)	Germany	Cohort	rt-fMRI NF	Y	4	40	None
Siniatchkin et al. (2000)	Germany	Cohort analytic	ERP-based NF	Y	10	72	None
Stokes and Lappin (2010)	USA	Cohort	Frequency NF	N	Varied *M* = 40 (30 NF + 10 pir-HEG)	30	Varied (3–24 months)
Vučković et al. (2019)	UK	Cohort	Frequency NF	Y	Varied *M* = 14 range (3–48)	25 to 30	None
Walker (2011)	USA	Cohort analytic	Frequency NF	Y	Varied M = 24 range (12–32)	30	None

*NR, not reported; NF, neurofeedback; RCT, randomized controlled trial; fMRI, functional magnetic resonance imaging; ERP, event related potential; LORETA, low-resolution electromagnetic tomography*.

### Synthesized Findings

#### Description of the Different NF Types and NF Protocols

A variety of NF types and protocols have been used for pain management. Most of them attempted to decrease brain activity hypothesized to be associated with the processing of nociceptive information (Siniatchkin et al., 2000; Emmert et al., 2014) and/or increase brain activity hypothesized to be inconsistent with pain information processing (Mathew et al., 1987; Jensen et al., 2014). Others aimed to normalize brain activity, relative to available normative data on brain activity (Koberda et al., 2013; Prinsloo et al., 2019). Here, we briefly describe the main characteristics of each type of NF used before discussing their effects on treatment outcomes.

We identified five different types of NF: four EEG-based and one fMRI-based. EEG-based NF asses and aim to modify the power of brain oscillation activity in different bandwidths from electrodes placed on the scalp. Brain oscillations are traditionally grouped in different bandwidths, expressed in cycles per second (Hz). The traditional bandwidths most often used for bandwidth classification, from slower to more rapid are: delta (δ, 0.5–4 Hz), theta (θ, 4–8 Hz), alpha (α, 8–13 Hz), beta (β, 13–30 Hz), and gamma (γ, 30+ Hz). Other bandwidths that are sometimes used in NF studies are most often subclassifications of these primary ones, such as low β (12–15 Hz) and high β (21-30 Hz) (Marzbani et al., 2016). Another common bandwidth used in NF studies is called “sensorimotor rhythm” (SMR) frequency (12–15 Hz). The SMR bandwidth is the same frequency as low β, but is a common frequency found in the sensorimotor areas of the cortex (Hoedlmoser et al., 2008).

##### Brain oscillation power-based NF

This type of NF that has been used most frequently in research in this area (Krigbaum and Wigton, 2014). This approach aims to increase or decrease the power of specific oscillation bandwidths as assessed from electrodes placed on different parts of the scalp. There is a large variety of protocols that have been used when treating patients with this procedure; in fact, we were unable to identify any studies that used the same NF protocol. That said, many of the protocols were quite similar. The protocols are often named based on the frequencies they seek to alter (e.g., an “alpha protocol” would be one seeking to alter – often increase – α power). This approach normally involves three electrodes: one for the active training site, one for the reference site, and one for ground. Some protocols using this approach are theory-based; that is, they intend to alter a frequency theorized to be associated with a behavioral outcome [e.g., increased α is associated with increased relaxation; (Hammond, 2011)]. Other protocols are data-based; that is, based on an initial quantitative electroencephalogram (qEEG) assessment of the patient that is then used to select the electrode positions and bandwidths to be targeted. Using the data-based approach, the participant is first administered a qEEG assessment to evaluate his or her unique EEG pattern, relative to a normative database. “Excesses” (power at bandwidths that are substantially greater than normative values) or “deficits” (power at bandwidths that are substantially lower than normative values) for any bandwidth activity at specific electrode sites are then identified, relative to healthy individuals. Once this assessment is conducted, an individualized treatment protocol is then designed to target any EEG “abnormalities” (i.e., deviations from the norm). The goal is to “normalize” the brain activity.

##### Surface Z-score NF and LORETA Z-score NF

To discuss the LORETA Z-score NF approach it is necessary to explain what LORETA imaging is. LORETA is a functional imaging procedure that seeks to estimate EEG bandwidth activity in deeper (intracranial) regions of the brain, based on data collected from surface electrodes (Pascual-Marqui et al., 1994, 2002). Similar to EEG data collected from specific electrodes, data from LORETA imaging can be compared with normative LORETA data, and then used to develop a treatment protocol (e.g., to reduce θ power in the thalamus, if a specific patient's pretreatment LORETA assessment indicates excessive thalamic θ). Alternatively, it is possible to simply determine that more (or less) power of a specific bandwidth at a certain intracranial site might decrease an individual's pain and, based on that information, to develop a protocol to use LORETA Z-score NF to alter activity in that bandwidth at that location. It is also possible to use a “normalizing” protocol in real time, such that the qEEG or LORETA-based data are compared to the norms directly, allowing to reinforce responses in the direction of the normative database. The use of qEEG and LORETA data in real time NF are commonly referred to as “surface Z-score NF” and “LORETA Z-score NF,” respectively (Wigton, 2013).

##### ERP-based NF

Event-related potential (ERP) assessments allow the study of stereotypical brain activity responses that occur at different specific time points following a specific stimulating cognitive, sensory or motor event (such as a response to an aversive stimuli; Luck, 2014). These time-locked brain responses to the aforementioned events are called components, which are believed to reflect the activity of postsynaptic potentials produced when thousands or millions of pyramidal neurons fire in synchrony while processing information (Sur and Sinha, 2009). ERP-based NF seeks to alter these components. One common ERP-based NF approach targets slow cortical potentials (SCP), which are slow event-related electrical shifts in the EEG of less than 1 Hz, that alternate between being electrically positive and negative (Wyckoff and Strehl, 2011; Krigbaum and Wigton, 2014). A distinctive component central to SCPs is the contingent negative variation (CNV), a negative potential that is recorded from the scalp during response anticipation, while the subject is anticipating and preparing for task performance (i.e., when they are told to press a button when a warning appears on the monitor). The aim of SCPs NF is to either increase or suppress the CNV by means of feedback, in order to regulate the excitation threshold (Strehl, 2009). Increased negativity is related to increased neural activity and a lower excitation threshold, whereas increased positivity is related to less neural activity and a higher excitation threshold (Strehl et al., 2006). Another ERP-based protocol that has been used for pain management targets changes in the amplitude of the N150-P260 complex, as this complex is sensitive to nociceptive stimulation (Miltner et al., 1988). The N150 is an early negative component that occur 150 milliseconds after the presentation of a stimulus, whereas the P260 is an early positive component that can be observed 260 milliseconds after the presentation of a stimulus.

##### Real-time fMRI NF

rt-fMRI NF allows patients to regulate brain activity in specific brain areas (including deeper areas of the brain) by targeting changes in the BOLD activity in the regions of interest. The most commonly used procedure in this type of NF involves an anatomical scan combined with a localizer task to identify the voxels of the region of interest to be trained (Sulzer et al., 2013; Thibault et al., 2018). Following this, the level of BOLD activity in the targeted area is fed back to the patient in order to facilitate their ability to increase or decrease that activity, as appropriate. The goal is to teach the individual to deliberately control the activation of the brain areas thought to be involved in pain perception and regulation.

### Evidence Regarding the Effects of EEG-Based NF

#### Brain Oscillation Power-Based NF

We identified 15 articles that evaluated the effects of brain oscillation power-based NF on pain and pain-related outcomes. In the first of these, a RCT was conducted to evaluate the efficacy of a SMR protocol in individuals with fibromyalgia (Kayiran et al., 2010). Participants were randomly allocated to either the NF group (*n* = 18) or an active control group (*n* = 18) receiving 10 mg of escitalopram per day for 8 weeks. The NF treatment was comprised of 20 30-min sessions aiming to increase SMR bandwidth activity assessed over the right-central area of the scalp (C4 in the international 10-20 system). In addition to assessing pain intensity, the authors assessed resting state EEG activity in the participants who received NF during an eyes-open condition at baseline, 2 weeks, 4 weeks (end of treatment), 8 weeks (1-month follow-up), 16 weeks (3-month follow-up) and 24 weeks (5-month follow-up) after treatment started. Although they found no changes in the mean amplitudes of resting state bandwidth power over time, there was a statistically significant decrease in the θ/SMR ratio at the end of the treatment, compared to baseline. Participants in both treatment conditions reported significant pre- to post-treatment reductions in pain intensity (measured with a 10-cm Visual Analog Scale), fatigue, anxiety and depression. The improvements were maintained at all the follow-up assessment points (i.e., up to 5 months after treatment started, or 4 months after treatment ended). In the NF group, the maximum reductions in both pain intensity and fatigue were reached at the 4th week of treatment (i.e., at the end of NF treatment), whereas in the active control group the greatest reduction in pain intensity was reported at the 8th week of treatment (i.e., at the end of active treatment for the control group). Moreover, the improvements in pain intensity, fatigue, anxiety and depression were significantly greater for the NF group than the control group at every assessment point. See Tables 3, 4 for a summary of the pain and brain activity outcomes for all the studies.

**Table 3:** Pre-treatment, post-treatment and follow-up pain intensity and frequency ratings.

**Authors (year)**	**Condition**	**Pain pre M (SD)/[SEM]**	**Pain post M(SD)/[SEM]**	**Pain follow-up M (SD)/[SEM]**
Kayiran et al. (2010)	E: NF	I: 8.9 (0.18)	I: 1.6 (0.21)	I (1 m): 1.9 (0,27) I (3 m): 2.4 (0.34) I (5 m): 2.6 (0.36)
	C: Escitalopram	I: 9.1 (0.23)	I: 4.7 (0.48)	I (1 m): 3.3 (0.27) I (3 m): 4.5 (0.34) I (5 m): 5.3 (0.30)
Prinsloo et al. (2018)	E: NF	I: 4.9 (0.35)	I: 2.7 (0.38)	I (1 m): 2.7 (0.51) I (4 m): 3.8 (0.48)
	C: WL	I: 4.4 (0.44)	I: 4.5 (0.35)	I (1 m): 4.6 (0.58) I (4 m): 4.6 (0.40)
Farahani et al. (2014)	E: NF	F (w): 4 (2.6)	F (w): 2.6 (1.77)	NA
	E: TENS	F (w): 5.4 (3.33)	F (w): 3.3 (1.68)	NA
	C: WL	F (w): 4.6 (4.43)	F (w): 4.4 (1.53)	NA
Stokes and Lappin (2010)	E: NF	F (m): 7.6 (5.1)	NA	F (3 m to 2 y): 2.9 (2.8)
Walker (2011)	E: NF	NR	F: 93% of participants > 50% reduction in migraine frequency.	NA
	C: Anti-migraine drug	NR	F: 8% of participants > 50% reduction in migraine frequency.	NA
Mathew et al. (1987)	E: NF	I: 6.2 (1.07)	I: 2.1 (1.23)	NA
	C: WL	I: 5.7 (1.71)	I: 3.9 (0.49)	NA
Caro and Winter (2011)	E: NF	NR	I: 39% reduction on average.	NA
	C: TAU	NR	I: No significant reduction on average.	NA
Jensen et al. (2007)	E: NF	I: 5.49 (2.24)	I: 3.2 (2.72)	NA
Hasan et al. (2015)^*^	E: NF	I: 7.3 (5.1)	I: 5.1 (1.46)	I: Reduced intensity compared to baseline but increased 1 to 2 points compared to last session.
Vučković et al. (2019)^T^^*^	E: NF	6.0	4.1	NA
Jensen et al. (2013a)	E: NF	I: 5.95 (1.7)	I: 5.4 (1.67)	I (3 m): 5.7 (1.90)
Jensen et al. (2013b)	E: NF	I: 4.61 (1.93)	I: 4.4 (2.09)	NA
	E: tDCS	I: 4.19 (2.02)	I: 3.9 (2.21)	
	E: Hypnosis	I: 4.27 (2.08)	I: 3.7 (2.16)	
	E: Concentration meditation	I: 4.44 (2.16)	I: 4.0 (1.97)	
	C: Sham tDCS	I: 4.39 (2.07)	I: 4.2 (2.02)	
Jacobs and Jensen (2015)	E: NF	All four participants reported significant pain intensity reductions.		
Jensen et al. (2016)	E: NF + Hypnosis	I: 5.3 (1.27)	I: 4.4 (0.71)	I (1 m): 4.0 (0.86)
	C: Relaxation + hypnosis	I: 5.2 (1.96)	I: 4.3 (1.9)	I (1 m): 4.3 (1.96)
Jensen et al. (2018)	E: NF + Hypnosis	I: 3.6 (1.17)	I (after NF): 2.6 (0.67) I (after hypnosis): 2.6 (1.20)	I (1 m): 2.4 (1.23)
	E: Mindfulness + Hypnosis	I: 3.8 (1.35)	I (after mindfulness): 2.8 (2.07) I (after hypnosis): 2.3 (2.42)	I (1 m): 3.3 (1.28)
	C: Hypnosis	I: 5.3 (1.57)	I (after hypnosis): 4.5 (2.61)	I (1 m): 4.5 (2.17)
Prinsloo et al. (2019)	E: NF	93% of the participants achieved significant reductions in pain intensity at either session 1 or 3.		
Koberda et al. (2013)	E: NF	All four patients reported reductions in pain intensity <50%.		
Miltner et al. (1988)	E: NF	6.4 (NR)	I (Increase N150-P260): 5 (1.62) I (Decrease N150-P260): 5.2 (1.63)	NA
Siniatchkin et al. (2000)	E: NF	I: 5.3 (1.4) F (m): 3.9 (2.5)	I:4.8 (2.3) F (m): 1.7 (1.8)	NA
	C: Healthy control	NA	NA	
	C: WL	I: 5.6 (1.8) F (m): 3.8 (3.6)	I: 6.0 (1.8) F (m): 4.0 (3.3)	
DeCharms et al. (2005)	*Individuals with c*hronic pain E: NF C: Autonomic biofeedback	44% reduction in pain intensity in the NF group, which was three times larger than for those in the biofeedback group.		NA
	*Healthy* individuals E: NF (rACC) C: 4 control groups with no feedback from rACC	In the experimental group, increasing or decreasing the BOLD activity in the rACC resulted in the noxious stimuli to be rated as more or less painful, respectively. The changes in pain intensity in the experimental were significantly larger than for any of the four control groups.		
Guan et al. (2015)	E: NF	I: 4.13 [0.55]	I (Up-training): increase in NRS scores of 1.8 [0.31] points. I (Down-training): decrease in NRS scores of 1.5 [0.33] points	NA
	C: Sham NF	I: 5.0 (0.52)	I (Up-training): increase in NRS scores of 0.1 [0.01] points. I (Down-training): decrease in NRS scores of 0.5 [0.22] points.	
Emmert et al. (2014)^*^	E: NF (lAIC)	I: 7.7 (1.20)	I: 6.0 (1.63)	NA
	E: NF (ACC)	I: 7.0 (1.15)	I: 6.2 (1.76)	
Rance et al. (2014a)	E: NF	None of the four conditions reported a significant decrease in pain intensity.		NA
Rance et al. (2014b)	E: NF	None of the two conditions reported a significant decrease in pain intensity.		NA

E, Experimental; C, Control; NF, neurofeedback; NA, Not assessed; NR, Not reported; TAU, Treatment as usual; WL, Wait-list control group; TENS, Transcutaneous electrical nerve stimulation; I, Intensity, F, Frequency; W, week; M, month; Y, year; [SEM] standard error of the mean; lAIC, Left anterior insular cortex; ACC, Anterior cingulate cortex; BOLD, blood oxygen-level dependent. ^*^ Pain intensity scores calculated from participants individual's data presented in the study. ^T^ Average pre- and post-session scores.

**Table 4:** Brain activity outcomes.

**Authors (year)**	**NF protocol**	**Effects in brain activity (pre- to post-treatment or during)**	**Association between brain activity change and pain improvements**
Kayiran et al. (2010)	↗ SMR at C4.	No changes in the mean amplitudes of EEG rhythms. A significant decrease in the θ/SMR ratio at the end of the treatment compared to baseline.	NA
Prinsloo et al. (2018)	Normalize EEG at several unreported scalp locations.	After treatment, the NF group significantly increased α activity and decreased β activity.	NA
Farahani et al. (2014)	↗ SMR, ↘θ and high β at T3 and T4.	NA	NA
Stokes and Lappin (2010)	NF, pir-HEG, hand-warming biofeedback. NF: normalize EEG at several scalp locations, mainly at: T3, T4, C3, C4, F3, F4, FP1, FP2, P3, P4.	NA	NA
Walker (2011)	↘ high β and ↗ 10 Hz activity at each electrode with excessive high β.	NA	NA
Mathew et al. (1987)	↗α at one or more unreported scalp locations.	The NF group showed an increase in the amount of time spent with a preponderance of α activity. In the NF group, there was no change in overall α amplitude. The wait-list control group did not evidence any significant brain activity change after treatment.	NA
Caro and Winter (2011)	↗ SMR, ↘θ and high β at Cz.	NA	NA
Jensen et al. (2007)	Tailored to each patient and adapted depending on patient's improvement. Normally started by ↗ SMR at T3 and T4.	NA	NA
Hasan et al. (2015)	First part: ↗α at Oz. Second part: combination of 4 protocols: A: ↗ SMR, ↘θ and high β at Cz. B: ↗α, ↘θ and high β at P4. C: ↗α, ↘θ and high β at C3. D: ↗α, ↘θ and high β at C4. *Placebo testi*ng protocol: Either prerecorded session or ↗α at Oz.	First part: All participants successfully increased at Oz, with no effect on pain intensity. Second part: All five participants decreased frontal θ during training. α power increased in the central cortex in four patients during training. Four patients decreased frontal high β during training. The largest long-term changes were in the high β band of the insular cortex, the cingulate cortex and the dorsolateral prefrontal cortex. Placebo testing protocol: During the placebo prerecorded session, the brain activity was not different from baseline. Participants were successful increasing α power at Oz, but this had no effect on pain intensity.	These patients that achieved a clinically meaningful reduction in pain intensity were the ones that successfully increased α power and to some degree, decreased β.
Vučković et al. (2019)	↗α, ↘θ and high β between C2 and C4.	With respect to baseline power: 9/15 participants significantly increased α power. 7/15 significantly decreased θ power. −6/15 participants significantly decreased high β power.	Brain activity changes after NF were partially associated with pain improvements. Eight of the 12 participants that achieved pain improvements successfully increased α during NF. Three of the remaining four participants who achieved pain improvements with NF but did not increase α, did achieve a significant decrease in θ, high β or both.
Jensen et al. (2013a)	3 protocols: A: ↗α and ↘β at T3 and T4. B: ↗ SMR, ↘θ and β at C3 and C4. C: ↗ SMR, ↘θ and β at P3 and P4.	Pre- to post-treatment decrease in θ and increase in α, that were no longer significant at 3-month follow-up. No changes in β activity.	NA
Jensen et al. (2013b)	↗α and ↘ high β at T3 and T4.	No significant pre- to post-session change in any of the five EEG bandwidths (δ, θ, α, β and γ).	There was no association between brain activity change with NF and pain changes.
Jacobs and Jensen (2015)	Tailored to each patient, but all received at some point a protocol involving ↗α, low β, ↘θ and high β. Several scalp locations used.	NA	NA
Jensen et al. (2016)	Before hypnosis: Increase θ (by ↗ 5–9 Hz and 8–11 Hz) at FP1 and F3. After hypnosis: ↗ low β, ↘γ, high β and θ at Cz.	NA	NA
Jensen et al. (2018)	↗θ at AFz.	There was no significant time effect in the NF group for any of the EEG bandwidths (δ, θ, α, β and γ).	NA
Prinsloo et al. (2019)	Normalize electrical activity in the Brodmann's areas 3, 4, 5, 13, 24, 32, and 33.	EEG changed toward EEG activity more representative of the normal population in all targeted Brodmann's areas but the 32.	Changes in the current source density in Brodmann's areas 24 and 33 accounted completely for the variance in pain changes with NF (*R^2^* = 1, *p* = 0.012).
Koberda et al. (2013)	Tailored protocols aimed at normalizing EEG activity.	The four participants evidenced changes toward a more normal brain activity pattern.	NA
Siniatchkin et al. (2000)	↗ and ↘ the amplitude of the SCPs at Cz.	Children with migraine were only able to decrease the amplitude of their SCPs; they were unable to increase cortical negativity. The control group of healthy children learned to both increase and decrease the amplitude of their SCPs.	No association between the change in the amplitude of the SCPs and the reduction of migraines.
Miltner et al. (1988)	↗ and ↘ the size of the N150-P260 complex at Cz.	Participants learned to increase and decrease the size of the N150-P260 complex. -Subjective pain intensity reports were slightly higher in the up-training condition compared to the down-training condition.	NA
DeCharms et al. (2005)	↗ and ↘ BOLD activity in the rACC.	The experimental healthy group learned to both increase and decrease BOLD activity in the rACC. The experimental group of patients with chronic pain learned to regulate BOLD activity in the rACC.	For the 6 patients with chronic pain that completed at least two training runs, there was a significant and strong association between the extent to which they were able to regulate BOLD activity in the rACC and pain intensity reductions (*r* = 0.9).
Guan et al. (2015)	↗ and ↘ BOLD activity in the rACC.	The experimental group was able to both increase and decrease BOLD activity in the rACC.	No association between the changes in BOLD activity and changes in pain ratings.
Emmert et al. (2014)	↘ BOLD activity in ACC. ↘ BOLD activity in lAIC.	Eight of the 14 participants were able to decrease the BOLD activity in the ACC. Nine of the 14 participants were able to decrease BOLD activity in the lAIC.	There were no differences in pain ratings between those who were able to decrease BOLD activity in lAIC and ACC and those who were not.
Rance et al. (2014a)	4 conditions: ↗ the BOLD activity in the rACC. ↗ the BOLD activity in the pInsL. ↘ the BOLD activity in the rACC. ↘ the BOLD activity in the pInsL.	Participants were able to increase BOLD activity in the pInsL and decrease BOLD activity in the rACC and pInsL.	NA
Rance et al. (2014b)	Increase the difference in activation levels between the rACC and pInsL. 2 conditions: [1] higher activation in rACC than in pInsL.	Participants were successful in achieving the training goals for the two conditions.	The achieved difference in activation between the rACC and the pInsL was not associated to changes in pain intensity ratings.
	[2] higher activation in pInsL than in rACC.		

*NA, Not assessed; NF, neurofeedback; EEG, electroencephalography; Hz, hertz; pir-HEG, passive infrared hemo-encephalography; SCPs, slow cortical potentials; ACC, anterior cingulate cortex; rACC, rostral anterior cingulate cortex; IAIC, left anterior insular cortex; plnsL, left posterior insula; BOLD, blood oxygen-level dependent*.

In another RCT, a sample of 71 cancer survivors with CIPN were randomly allocated to the NF group (*n* = 35) or to a wait-list control group (*n* = 36) (Prinsloo et al., 2018). A qEEG was conducted and used to develop patient-specific NF protocols to normalize EEG-assessed oscillation power. The NF treatment consisted in 20 45-min sessions. The average pain intensity and pain interference ratings for the NF group were significantly lower at the end of the treatment compared to the wait-list control group; these differences were still statistically significant at 1-month and 4-month follow-up assessment points. Although there was also a significant difference in fatigue ratings between groups at the end of treatment, these differences were no longer statistically significant at 1-month and 4-month follow-up. There were no significant between-group differences in sleep quality or sleep disturbances at any assessment point. Results showed that brain activity, that is, the EEG frequencies targeted in the scalp positions chosen by the protocol, changed significantly from pre- to post-treatment toward a more “normal” EEG activity and that it was significantly different for the NF group compared to the waitlist group. Specifically, the NF group showed a significant increase in α relative power and a significant decrease in β relative power as averaged over all the electrodes.

Another RCT compared the efficacy of NF and transcutaneous electrical nerve stimulation (TENS) in a group of 45 healthcare practitioners with primary headaches (Farahani et al., 2014). Participants were randomly allocated to either a NF group (*n* = 15), a TENS group (*n* = 15) or a waitlist-control group (*n* = 15). The NF treatment consisted of 20 30-min sessions and aimed to increase SMR and decrease θ and high β over the right and left temporal cortex (T3 and T4 in the international 10–20 system). Both the NF and TENS groups experienced significant reductions in headache frequency compared to the waitlist-control group. However, the NF group achieved a significantly greater reduction in headache frequency than the TENS group.

In an uncontrolled study (Stokes and Lappin, 2010), 37 patients with migraine were treated with a combination of NF, passive infrared hemo-encephalography (pIR-HEG; a form of neurofeedback based on thermal outputs in response to changes in blood flow dynamics rather than brain electrical activity Carmen, 2004), and thermal biofeedback (i.e., a type of biofeedback that aims to change body temperature). The treatment consisted of an average of 40 sessions and included an average of 30 frequency-based NF sessions and an average of 10 pIR-HEG or hand-warming biofeedback sessions. NF training aimed to reduce the amplitude of the frequencies which were assessed at baseline and determined to be “excessive;” that is, treatment was tailored to each participant and was not standardized. The scalp positions where NF was conducted were primarily 5 sets of homologous sites (including over the prefrontal, frontal, temporal, central and parietal areas; FP1-FP2, F3-F4, T3-T4, C3-C4, and P3-P4 in the international 10–20 system). Compared with baseline scores, patients reported a significant reduction in the number of migraines per month at follow-up (a post-treatment assessment was not conducted), which was conducted three months to two years after the end of the treatment.

Walker studied the effects of NF as a treatment for recurrent migraine headaches (Walker, 2011). Of the 76 individuals entering the study, 46 chose to follow the NF treatment and 25 chose to remain with anti-migraine medication (the specific medication used by the study participants was not reported). The qEEG analysis at baseline showed an excess of power in the high β frequency band at a number of electrode sites – excesses that were most pronounced in the frontal, central and parietal regions. The NF protocol consisted in five 30-min sessions targeting a reduction in high β activity and an increase in 10 Hz activity at each electrode where an excessive high β activity had been identified. At post-treatment, 98% and 32% of the participants in the NF and control condition reported reductions in headache frequency, respectively. Specifically, in the NF group, 54% experienced a complete cessation of migraine headaches, 39% experienced a reduction in migraine headaches greater than 50, and 4% experienced a reduction of <50%. In the control group, none of the participants experienced a complete cessation of migraine headaches, 8% experienced a reduction in migraine headaches greater than 50, and 20% experienced a reduction of less than 50%.

The oldest study included in this review (Mathew et al., 1987) assessed the efficacy of NF as a treatment for eight individuals with tension-type headache compared to a waitlist control group (*n* = 4). The NF participants received 20 30-min sessions of a protocol aiming to increase α assessed from one or more (unreported) electrode sites. The treatment group reported a significant increase in the amount of time spent with a preponderance of α activity, but not in its overall amplitude. The NF group also reported statistically significant reductions in pain intensity and anxiety from pre- to post-treatment. The waitlist control group, on the other hand, did not evidence any significant changes in brain activity, pain intensity or anxiety from pre- to post-assessment.

Caro and colleagues conducted an uncontrolled study assessing the use of NF to reduce attention difficulties and somatic symptoms in patients with fibromyalgia (Caro and Winter, 2011). Fifteen patients were treated with NF and compared with a historical control group comprised of 63 individuals receiving standard medical care. The NF group received 58 sessions on average (ranging from 40 to 98) aiming to increase SMR oscillation power, while inhibiting both θ and high β oscillations at the same time. The training electrode was placed over the center of the scalp (Cz in the international 10–20 system). The NF group reported significant mean reductions in global pain and fatigue severity (39 and 40%, respectively). The 63 control participants did not report any significant improvements in either outcome variable.

Another study reported on changes after a single session of NF in 18 individuals with CRPS-I participating in a 20-day multidisciplinary treatment program (Jensen et al., 2007). The treatment protocol used varied over the course of each 30-min session, and was tailored to each patient, depending on their reports of pain reduction (or not) as the session progressed. For example, if training at a specific site to increase the power of a specific bandwidth was associated with improvements, that training continued. Training usually began by reinforcing SMR activity at sites over temporal areas (T3 and T4 in the international 10–20 system) to “stabilize” brain activity. If the patient reported no improvement with this protocol, different electrode sites or training frequencies were used until (and if) the patient reported improvements. Participants reported an average pre- to post-session reduction of 2.3 points in pain intensity (on a 0–10 Numerical Rating Scale) of their primary pain. Half of the participants reported a pain intensity reduction that was clinically meaningful, that is, a reduction of 30% or more from pre- to post-session (Rowbotham, 2001).

A pilot study (Hasan et al., 2015) aimed to investigate the potential mechanisms underlying NF efficacy to treat central neuropathic pain in seven patients with chronic paraplegia. Four patients received 40 sessions, one received 20 and two received only three sessions. The first 10 min of the NF treatment aimed to increase α at occipital regions (Oz in the international 10-20 system) with a goal of increasing general relaxation. The remainder of the NF training session had a goal of pain reduction. In this second component of each training session, each patient received a combination of one of four different protocols (all in a 30- to 35-min period), depending on their response to each. Protocol A reinforced SMR and suppressed θ and high β assessed from the central area of the scalp (Cz in the international 10–20 system). Protocol B reinforced α and suppressed θ and high β from an electrode placed over the right parietal area (P4 in the international 10–20 system). Protocol C reinforced α and suppressed θ and high β from an electrode placed over the left central area (C3 in the international 10–20 system). Protocol D reinforced α and suppressed θ and high β at from an electrode placed over the right central area (C4 in the international 10–20 system). It is important to note that the α range targeted in this study was slightly higher than usual, that is, 9–12 Hz instead of the general 8–12 Hz, as lower α frequencies have been found to be associated with central neuropathic pain (Boord et al., 2008). Also, each participant received two “placebo” sessions at some point between sessions 10 and 20 (the specific sessions that were “placebo” sessions differed for each participant), with the goal of testing for placebo responses. One placebo protocol “fed back” pre-recorded data from a different NF session, and the other provided feedback aiming to increase α at the occipital area (Oz in the 10–20 system). Both placebo protocols were hypothesized to not have any impact on pain. Resting state EEG in both open eyes and closed eyes conditions and sLORETA imaging (a newer and more accurate LORETA) was recorded before and after treatment. In addition, the researchers assessed and recorded EEG activity before and during NF training. All participants received a different number of sessions of each protocol, and the sequence of protocols used also differed for each patient and changed depending on their initial response. The five patients that received at least 20 sessions reported statistically significant pre- to post-treatment reductions in pain intensity; four (80%) reported pain reductions that were clinically meaningful (>30%). The patients that achieved clinically meaningful reductions in pain intensity were the ones that successfully increased α power and, to some degree, decreased high β power. At one-month follow-up assessment the participants who reported reductions in pain still reported lower pain intensity, relative to baseline, although they also reported an increase in pain intensity of one to two points (on a 0–10 scale), relative to baseline. Additionally, regarding pre- to post-session effects, protocols C and D were associated with the greatest reductions in pain intensity, although three patients had strong muscle spasms with protocol C. Protocol B yielded a moderate reduction in pain intensity whereas protocol A did not decrease pain intensity for any of the patients. Also, in the two sessions used to test for placebo effects, participants successfully increased α power at the central occipital area (Oz in the international 10–20 system), but this had no impact in pain intensity. The largest long-term changes were in the high β band of the insular cortex, the cingulate cortex and the dorsolateral prefrontal cortex, assessed via sLORETA.

Another study conducted by the same research team tested the use of self-administered NF to treat central neuropathic pain in 15 patients with chronic SCI (Vučković et al., 2019). Participants were offered up to four training sessions at the hospital before they had to self-administer the treatment at home. They were instructed to use NF on demand but at least once a week for two months, and to record pain intensity before and after each session. The NF session protocol consisted in reinforcing α power and suppressing θ and high β power as measured at a central site (specifically between C2 and C4 in the international 10–20 system). As in the previous study conducted by the same research team, the α range targeted was slightly higher than usual (i.e., 9–12 Hz). Each session lasted 30 to 35 min. In total, participants received or self-administered an average of 14 sessions, ranging from 3 to 48 sessions. Statistically significant pre- to post-session improvements in average pain intensity were found in 12 of the 15 participants, with eight participants achieving clinically meaningful reductions in each session on average. With respect to brain activity changes, each NF session was preceded by 2-min baseline EEG recording in the eyes-opened condition. Of the 15 participants, nine significantly increased α power with treatment, whereas seven and six participants significantly decreased θ and high β power, respectively. These changes were partially associated with pain improvements. Specifically, eight of the 12 participants that achieved pain improvements successfully increased α during NF. Three of the remaining four participants who achieved pain improvements with NF but did not increase α, did achieve a significant decrease in θ, high β or both.

Another study tested the efficacy of three different NF protocols in 10 individuals with SCI and chronic pain (Jensen et al., 2013a). Each individual received 4 sessions of each of the following protocols in random order. Protocol A reinforced α and suppressed β activity measured from electrodes at the temporal sites frequently used in NF treatment for pain management (i.e., T3 and T4 in the international 10–20 system). Protocol B reinforced SMR activity and suppressed β and θ power assessed from electrodes at central sites (C3 and C4 in the international 10–20 system). Protocol C reinforced SMR activity and suppressed β and θ power at parietal sites (P3 and P4 in the international 10–20 system). There were similar pre- to post-session reductions in pain intensity for all three protocols. However, statistically significant pre- to post-treatment (i.e., after the 12 sessions) reductions were not found in average pain intensity. In addition, there were not statistically significant pre- to post-treatment improvements in fatigue, sleep quality and pain interference. The investigators also assessed and reported resting EEG in eyes closed condition at pretreatment, post-treatment and 3-month follow-up. In line with the protocols, there were both an increase of α power and a decrease in θ power from pre- to post-treatment. These changes in α and θ power were not sustained and were no longer different from baseline levels at the 3-month follow-up. β power did not change significantly over time, despite the fact that all three protocols aimed to decrease it.

Another study (Jensen et al., 2013b) assessed the effects of a single 20-min session of four different interventions [NF, hypnosis, concentration-meditation and transcranial Direct Current Stimulation (tDCS)] on pain intensity in thirty patients with SCI and chronic pain, compared to a tDCS sham procedure. Each intervention session took place in a different day. The NF session protocol consisted in reinforcing α and suppressing high β power measured at right and left temporal sites (T3 and T4 in the international 10–20 system). In addition, resting state EEG was recorded for 10 min in eyes closed before and after each of the five procedures. Neither pain intensity nor EEG activity in any of the five bandwidths (i.e., δ, θ, α, β, and γ) changed significantly after a single session of NF. Also, the associations between changes in EEG power at the different bandwidths and changes in pain intensity were not significant.

Jacobs and Jensen (Jacobs and Jensen, 2015) published a case series reporting the use of NF as a treatment for four individuals with a variety of chronic pain problems. The first patient was a 19-year-old girl with abdominal pain. She received 41 NF sessions. The second patient was a 56-year-old woman with migraine headaches who received 32 sessions. The third patient was a 14-year-old young man with chronic testicular pain who received 22 NF sessions, and the fourth patient was a 47-year-old man with severe gastrointestinal pain who received 26 sessions of NF treatment. The treatment protocols were tailored for each patient based on standard practice recommendations for addressing the presenting problems of the patients. Given the common practice of rewarding increases in α and low β power for chronic pain management, all the patients received training that involved these components for at least some of the sessions. Specifically, at some point, they all received a protocol that involved rewarding increases in α and low β power and decreases in θ and high β power. A number of electrode positions were used as training sites, with the goal of identifying the sites and protocols that would be most effective for each patient. All four patients achieved clinically meaningful reductions in pain intensity or pain frequency at some point during treatment, although one of the patients reported that his pain intensity returned to baseline levels by the end of the treatment.

Two pilot studies were conducted to explore the possibility that NF might be used for enhancing the effect of hypnosis for chronic pain management in individuals with multiple sclerosis. In the first of these (Jensen et al., 2016), participants were randomly allocated to receive five sessions of self-hypnosis (one face-to-face session and four prerecorded sessions), preceded by either four 30-min sessions of NF (*n* = 10) or four 20-min sessions of relaxation training, which served as a control group (*n* = 9). After each session, all the individuals received one self-hypnosis session. The NF protocol aimed to increase θ power by reinforcing slow wave power (5–9 and 8–11 Hz) at frontal sites (FP1 and F3 in the international 10–20 system), based on evidence suggesting that higher levels of θ power are associated with greater response to hypnosis (Jensen et al., 2015). These investigators had a concern that an excess of θ power might result in negative effects, given the association between θ activity and having a diagnosis of attention deficit disorder (Arns et al., 2013). To address this possibility, after each hypnosis session, the participants received 10 additional minutes of a NF protocol aiming to reverse any enhanced θ with a protocol reinforcing low β while inhibiting γ, high β, and θ at a central site (Cz in the international 10–20 system). The participants who received the hypnosis treatment preceded by either the NF or relaxation treatment reported statistically significant reductions in average pain intensity, pain interference and fatigue severity. In the group receiving NF treatment, participants reported larger decreases in average pain intensity from pre- to post-treatment and from pretreatment to 1-month follow up, compared to the participants receiving the relaxation treatment. No differences between the NF and relaxation groups were found regarding improvements in pain interference or fatigue severity.

In the second study (Jensen et al., 2018), individuals with multiple sclerosis and either chronic pain, chronic fatigue or both pain and fatigue, were randomly allocated to receive five sessions of self-hypnosis (one face-to-face session and four prerecorded sessions), preceded by either six 30-min sessions of NF (*n* = 12), six 30-min sessions of mindfulness meditation (MM; *n* = 10) or no intervention (*n* = 10). After this, all participants received one face-to-face hypnosis session, and then four prerecorded hypnosis sessions (recorded by the same clinicians who provided the single face-to-face hypnosis session), targeting pain reduction, fatigue reduction, or both, depending on the presenting problem(s) of the participants. The NF group received in addition four sessions of NF immediately before the recorded hypnosis treatment sessions, and the MM group received an additional four sessions of MM immediately before the recorded hypnosis treatment sessions. Therefore, the NF and MM groups received 11 sessions in total (six NF or MM sessions alone, one face-to-face hypnosis session, and then four “combined” NF with hypnosis or MM with hypnosis sessions), and the control group received five sessions in total (the single face-to-face hypnosis session and four pre-recorded hypnosis sessions. The NF protocol reinforced an increase in θ power at the frontal midline region of the scalp (AFz in the international 10–20 system). Participants in all three conditions reported statistically significant reductions in pain intensity from pretreatment to 1-month follow-up, which were the highest for the NF group. Both the NF and MM groups reported similar significant pain intensity reductions with six sessions of each treatment alone. At 1-month follow-up, the NF group had maintained the gains made during treatment, whereas the pain intensity ratings in the MM group returned to baseline levels. Fatigue severity ratings improved similarly for the three groups, with a small decrease from baseline to before the hypnotic treatment and an additional decrease after the hypnotic treatment. Nevertheless, fatigue severity increased slightly from post-treatment to follow-up. With respect to the secondary outcomes (sleep disturbance, pain interference and depression), only the NF group reported significant improvements from pretreatment to 1-month follow-up. EEG data were recorded for both the NF and MM groups at baseline, after the first six sessions (pre-hypnosis) and at the last NF or MM session (post-treatment). Although there were some differences in the mean amplitudes of the five EEG bandwidths from baseline to pre-hypnosis or from pre-hypnosis to post-treatment, there was no significant time effect for neither the NF nor the MM groups.

#### Surface Z-Score NF and LORETA Z-Score NF

Prinsloo and colleagues conducted an exploratory study to assess the use of LORETA Z-score NF (i.e., with a goal toward normalizing brain activity) to treat pain in patients with head and neck cancer undergoing radiation therapy (Prinsloo et al., 2019). In this study, pain intensity and resting eyes-open EEG activity was measured at three time points: baseline (i.e., before starting radiation therapy), after starting radiation therapy and when and if patients reported a pain intensity score of 4 or higher, and after the NF treatment. Pain intensity was also assessed and reported before and after NF sessions 1 and 3. Fourteen patients received one to six 20-min sessions of LORETA Z-score NF targeting a normalization of the activity in the Brodmann's areas number three, four, five, 13, 24, 32, and 33, in real time. As reported by the investigators, 14 patients received one or more sessions, 12 received at least three sessions and five received six NF sessions. Significant pre- to post-session reductions in pain intensity was reported by 93% of the participants at either session one (n=9), with an average mean reduction of 2.1 points (SD = 1.54; on a 0–10 NRS scale) or session three (*n* = 8), with an average reduction of 1.13 points (SD = 0.35; it was not clear based on the data presented by the investigators how many of these participants reported significant pain reductions in both sessions). With respect to brain activity changes, there was a change toward normality in the current source density of all targeted brain areas but one (i.e., Brodmann's area 32). Interestingly, regression analysis found that changes in the current source density in Brodmann's area 24 accounted for ~92% of pain variance, and current source density in Brodmann's area 33 accounted for the rest. Specifically, lower levels of current source density in Brodmann's area 24 and higher levels of current source density in Brodmann's area 33 were significant predictors of pain intensity.

Another case series (Koberda et al., 2013) reported the use of both 19-channel Surface Z-score and 19-channel LORETA Z-score NF to decrease pain in four patients with different pain problems. The first patient had neuropathic pain and received 65 sessions. At the initial assessment, his qEEG showed an excess of β activity at temporal locations whereas LORETA imaging showed an excess in θ and β activity at the left insular cortex. The second patient had chronic pain associated with depression and received 25 sessions. Her initial qEEG showed an excess of δ and β power in frontal and central areas, and the LORETA imaging showed “dysregulation” in the anterior cingulate cortex. The third patient had both postherpetic neuropathy and sensory motor polyneuropathy, and received 45 sessions. His qEEG showed an excess of δ power in frontal areas, and the LORETA imaging showed “dysregulation” in the left insular cortex. The fourth and final patient had trigeminal neuralgia and received 10 sessions of NF. Her qEEG showed an excess of δ and θ power in fronto-temporal areas and an excess of β in frontal areas, whereas the LORETA imaging showed “dysregulation” in the left insular cortex. The investigators did not specify the number of sessions that each patient received of each treatment approach (i.e., surface Z-score or LORETA Z-score NF). Compared with the pre-treatment pain levels, all the patients reported substantial reductions in pain intensity, ranging from 50% reduction to complete remission. With respect to brain activity changes, and whether assessed with qEEG or LORETA, all patients evidenced changes in the direction of more normal brain activity patterns over the course of treatment.

#### ERP-Based NF

Two studies used ERP-based NF to modulate pain: one was a clinical study whereas the other was an experimental study with laboratory induced pain.

#### Clinical Pain Study

The first study (Siniatchkin et al., 2000) was a controlled trial that examined the efficacy of Slow Cortical Potentials NF in a small sample (*n* = 10) of children with migraine without aura. Participants in this study were compared with two control groups: a wait-list control group of children with migraines (*n* = 10) and a control group of healthy children who also received the NF treatment (*n* = 10). This latter control group was used to compare the ability to self-regulate slow cortical potentials in children with migraine compared to healthy children. The NF protocol was conducted with brain activity measures from the central region of the scalp (Cz in the international 10–20 system) and consisted in two different tasks that were trained during the same session: each task was to either increase or decrease the amplitude of the SCPs. Additionally, EEG was recorded at frontal and central sites (Fz and Cz in the international 10–20 system). Children in the treatment group and in the healthy control group were able to control the amplitude of their SCPs after the 10 sessions. However, the group of children with migraine was only able to decrease cortical negativity (i.e., decrease the amplitude of their SCPs). After 10 sessions, the treatment group showed significant reductions in the number of days with migraine per month; effects that were not found in the wait-list control group. There was no association between the extent of decrease in the amplitude of the SCPs with NF and the reduction in the number of days with migraine.

#### Laboratory Induced Pain Study

The second study aimed to test whether it was possible to modify pain intensity via increasing the ability to alter the N150-P260 complex evoked by aversive stimulation (Miltner et al., 1988). In this study, 10 otherwise healthy male individuals underwent a single 120-min experimental session. First, the individual's pain threshold and the amount of noxious stimulation required for the participant to experience a pain intensity at 20% above his or her pain threshold were measured. Then, the baseline ERPs and subjective pain intensity in response to the simulation with an intensity of 20% above the threshold were measured. The last part of the session was devoted to the NF training in the form of two different tasks when presented with the same noxious stimulation used at baseline (i.e., 20% above threshold): one in which the subjects were reinforced for increasing the size of the N150-P260 complex and one in which they were reinforced for decreasing the size of this complex. Both tasks were randomly presented during the session. EEG was recorded at central areas of the scalp (i.e., Cz according the international 10–20 system), where the NF intervention was conducted. With respect to brain activity, the subjects were able to learn to alter the size of the N150-P260 complex consistent with the training. Also, pain intensity reports were different in the up-training and down-training conditions; when presented with identical noxious stimuli, those in the up-training condition reported slightly higher pain intensity reports than those in the down-training condition. Despite the differences in pain intensity reports between both conditions, however, the decrease after the whole session in pain intensity ratings was not statistically significant.

### Evidence Regarding the Effects of fMRI-Based NF

To date, five studies have evaluated the efficacy of rt-fMRI NF to modulate pain: two were clinical studies whereas the other three were experimental studies with laboratory induced pain.

#### Clinical Pain Studies

DeCharms and colleagues tested whether it was possible for individuals to learn to control brain activation in the rostral anterior cingulate cortex (rACC) in a single session of rt-fMRI NF (DeCharms et al., 2005). This study used seven groups. Of these, two were experimental groups that received the rt-fMRI NF and five were control groups. The first experimental group, which was comprised of eight healthy individuals, was compared to four healthy control groups (three of them had eight individuals and one had four individuals) that underwent similar procedures but without valid feedback from rACC (i.e., training using sham rt-fMRI data belonging to another subject recorded session, or training using rt-fMRI data from a brain area other than the rACC). These four control groups were used to determine if the effects of the rt-fMRI NF were due to the ability to modulate the activation in the rACC rather than due to non-specific (i.e., placebo) effects. The second experimental group, which was comprised of eight patients with chronic pain, was compared to a control group of four patients with chronic pain that were trained with autonomic biofeedback. The rt-fMRI NF protocol consisted of training runs (i.e., a specific training period within a training session) in which participants were asked to both increase and decrease BOLD activity in the region of interest within the rACC, hypothesized to be an important area underlying the experience of pain. Each training run lasted 13 min and was comprised by five 60-second increase cycles and five 60-s decrease cycles. A thermal noxious stimulus was presented for 30 s to the healthy participants only in each cycle. All the healthy subjects went through a localizer scan, three training runs and a posttest scan, whereas, patients with chronic pain also had the localizer and posttest scan but could choose the number of training runs they were willing to do. Thus, four patients had three training runs, two patients had two training runs and two patients had one training run. After each training run, all study participants were asked to report pain intensity.

The experimental healthy group learned to modulate the BOLD activity in the rACC, whereas the control groups did not. The experimental healthy group learned to both increase and decrease BOLD activity in the rACC, affecting pain perception differently. That is, noxious stimuli presented when subjects were trying to increase BOLD activity in the rACC were rated as significantly more painful than when subjects were trying to do the opposite; that is, to decrease BOLD activity in the rACC activation. The control over pain intensity achieved by the healthy experimental group (who trained with valid feedback from rACC) was significantly larger than for any of the four healthy control groups (who underwent similar training but without valid feedback from rACC). With respect to the experimental group of patients with chronic pain, they reported a 44% pre- to post-session decrease in pain intensity. There was a strong association between the level of control over the BOLD activity in the rACC achieved by the patients with chronic pain after rt-fMRI NF and the change in pain ratings (*r* = 0.9, *p* < 0.01). Also, the pain intensity reductions in this group were three times greater than those reported by participants who received the autonomic biofeedback intervention.

A more recent study evaluated the effects of a single session of rt-fMRI NF to teach voluntary control over activation in the rACC (Guan et al., 2015). The participants in this study had postherpetic neuralgia, and were randomly allocated to either an experimental group, which received real information from the rACC, or to a control group, which received sham information from a different brain region (i.e., the posterior cingulate cortex). In this experiment, both the experimental (*n* = 8) and the control (*n* = 6) groups were reinforced at different times for increasing and decreasing activation in the respective regions of interest. The experimental group was able to both up- and down-regulate BOLD activity in the rACC significantly better than the control group, suggesting that rACC activity may be more amendable to control than activity in the posterior cingulate cortex. Moreover, the experimental group achieved significantly greater changes in pain intensity compared to the control group. In the up-regulation condition, pain intensity ratings increased 1.8 and 0.1 (on a 0–10 scale) for the experimental and control groups, respectively. In the down-regulation condition, pain intensity ratings decreased 1.5 for the experimental group and 0.5 in the control group. However, the associations between changes in BOLD activity and changes in pain intensity for either the up- and down-regulation conditions were not statistically significant.

#### Laboratory Induced Pain Studies

Emmert et al. (2014) assessed the use of a single session of rt-fMRI NF in healthy individuals targeting two different regions hypothesized to be associated with the processing of pain information: the ACC and the left anterior insular cortex (lAIC). Both groups were first asked to participate in a localizer task with noxious heat stimulation to establish the specific pain-sensitive target region in the AIC or ACC for each participant. Next the NF training was conducted, during which participants received feedback to decrease the BOLD activity during pain stimulation in the brain area identified during the localizer task for that participant. Over half of the participants in each group were able to successfully decrease BOLD activity in either the ACC or lAIC. Both the lAIC (*n* = 14) and ACC (*n* = 14) groups significantly reduced pain ratings in the feedback task compared to the localizer task. Moreover, there was no significant difference in the reduction of pain intensity between the lAIC and the ACC groups, nor there was a significant difference in pain ratings between those who successfully decreased BOLD activity and those who did not.

The final two studies were conducted by a single research team and used similar procedures. Both studies included 10 healthy individuals. The investigators conducted an anatomical scan, a baseline run, and 24 training runs over four consecutive days. Each of the training runs was comprised of six regulation phases (where the individuals received electrical noxious stimulation along with rt-fMRI NF training) and six non-regulation phases (where participants engaged in mental arithmetic tasks).

The first study (Rance et al., 2014a) aimed to evaluate the effect of separately increasing and decreasing the BOLD activity in the rACC and left posterior insula (pInsL) on pain intensity. The study had four conditions: increase BOLD activity in rACC, decrease BOLD activity in rACC, increase BOLD activity in pInsL and decrease BOLD activity in pInsL. Three of the conditions (all except the condition that aimed to increase activity in the rACC) resulted in brain activity changes in the intended directions. However, none of the four conditions resulted in significant changes in pain intensity ratings.

In the second study, the investigators (Rance et al., 2014b) aimed to assess the effect of disrupting a part of the pain processing network by training participants to increase the *difference* in activation levels between two brain regions: the rACC and pInsL. Participants received rt-fMRI NF training with the goal to achieve two states: one where the activation of the rACC was higher than the activation of the pInsL, and a second state where the activation of the pInsL was higher than the activation of the rACC. Although the participants were successful in achieving the training goals, pain intensity ratings did not change significantly from the first to the last training trial.

### Risk of Bias

The details of the quality ratings according to the Quality Assessment Tool for Quantitative Studies are presented in Table 5. It is noteworthy that none of the studies received a “strong” rating for the components of selection bias and confounders. All but two of the studies were rated as either “strong” or “moderate” in study design. Most (*k* = 17, 71%) of the studies were rated as weak in the blinding component, and just one study (4%) was double-blinded (i.e., it received a “strong” rating for the blinding component). Seventeen studies (71%) used reliable and valid measures to assess outcomes and 14 studies (58%) were rated as “strong” with respect to withdrawals and drop-outs.

**Table 5:** Quality ratings for the included studies.

**Authors, year**	**Selection bias**	**Study design**	**Confounders**	**Blinding**	**Data collection methods**	**Withdrawals and drop-outs**
Caro and Winter (2011)	3	2	N/A	3	2	3
DeCharms et al. (2005)	2	2	3	3	1	1
Emmert et al. (2014)	3	1	2	3	1	1
Farahani et al. (2014)	2	1	2	3	1	1
Guan et al. (2015)	3	1	2	1	1	1
Hasan et al. (2015)	3	2	N/A	3	1	2
Jacobs and Jensen (2015)	3	3	N/A	N/A	3	N/A
Jensen et al. (2018)	2	1	2	3	1	1
Jensen et al. (2013a)	3	2	N/A	3	1	1
Jensen et al. (2016)	3	1	3	3	1	1
Jensen et al. (2007)	3	2	N/A	3	2	1
Jensen et al. (2013b)	2	2	N/A	2	1	1
Kayiran et al. (2010)	3	1	3	2	1	1
Koberda et al. (2013)	3	3	N/A	N/A	3	N/A
Mathew et al. (1987)	3	1	3	3	2	2
Miltner et al. (1988)	2	2	N/A	3	1	1
Prinsloo et al. (2019)	3	2	N/A	3	1	2
Prinsloo et al. (2018)	2	1	2	3	1	2
Rance et al. (2014a)	3	2	2	3	1	1
Rance et al. (2014b)	3	2	2	3	1	1
Siniatchkin et al. (2000)	3	1	2	2	1	3
Stokes and Lappin (2010)	3	2	N/A	3	3	1
Vučković et al. (2019)	3	2	N/A	N/A	1	2
Walker (2011)	3	2	N/A	3	3	3

*Strong (1) Moderate (2) Weak (3); confounders and blinding components were not assessed for studies without control group or for case-series; withdrawals/drop-outs component was not assessed for case-series*.

## Discussion

In this review we summarized the available evidence regarding the efficacy of NF as a treatment for pain and its effects on pain-related brain activity. To our knowledge, this is the first review to systematically summarize the use and effects of NF as an intervention for any type of pain and pain-related outcomes.

### NF Protocols Studied

The first aim of this review was to describe the different types of NF and NF protocols that have been used in pain research and how NF has been used for pain management. Most of the 24 studies that were included and reviewed were EEG-based and focused mostly on adults with migraines or headache and other chronic pain conditions, such as fibromyalgia or cancer-related pain. Of the five types of NF that we identified and described, brain oscillation power-based NF was evaluated the most often.

Within each type of NF studied, the specific protocols used varied from study to study. Although some NF protocols shared some features, no two studies used the exact same protocol. To the extent that several high-quality clinical trials are needed to draw conclusions regarding the efficacy of a clinical intervention, the lack of consistency in the NF protocols studied means that the field has not advanced enough to be able to draw strong conclusions regarding the efficacy of specific NF protocols for pain management.

### Efficacy of NF

The second aim of the study was to summarize the evidence regarding NF and different NF protocols for modulating pain and improving pain-related outcomes. As a whole, and given the generally positive results in the studies reviewed, the findings indicate that NF procedures have the potential for reducing pain and improving other outcomes in individuals with chronic pain. Most of the studies reviewed found significant pre- to post-treatment improvements in pain intensity and/or pain frequency, with some of these improvements being maintained at follow-up (when follow-up was evaluated). Also, most of these studies found significant improvements in other pain-related variables such as fatigue, sleep problems/sleep quality, anxiety, depression, and pain-related interference. NF was also found to enhance the effects of hypnosis for chronic pain management and to reduce the perception of experimentally induced pain in healthy individuals.

However, and as alluded to previously, the high level of protocol heterogeneity and the heterogeneity in the characteristics of the samples studied do not allow us to draw conclusions regarding the efficacy of NF types and specific NF protocols. That said, there were some patterns in the study findings that could be used for hypothesis generation for future research. For example, the brain oscillation power-based NF protocols often included some combination of protocols that increased α and SMR power, and decreased β and θ power. Another commonly used protocol was to tailor NF treatment to each individual participant based on their baseline qEEG assessment, with a goal of bringing their qEEG in line with normative values. Most of these studies found positive results for the NF interventions evaluated. These preliminary findings raise the possibility that the beneficial effects of NF may be due to (1) NF's effects on the power of one or more specific bandwidths or (2) NF's ability to normalize bandwidth power across the spectrum. The need for more research in this area is discussed in more detail in the next section.

### The Mechanisms That Underlie NF Treatment

As mentioned previously, in the context of pain treatment, NF aims to change brain activity that is thought to underlie or influence the experience of pain (Ibric and Dragomirescu, 2009). The third aim of the current review was to determine the level of evidence regarding the effect of NF training on targeted brain activity, and the associations of these with improvements in pain outcomes. Unfortunately, almost a third of the studies included in the review did not assess changes in brain activity. Moreover, those studies that did include some measure of brain activity studied different domains of brain activity. For example, some studies evaluated whether there were any brain activity changes *during* a training session or training sessions, whereas others evaluated pre- to post-treatment changes in resting state activity.

An important question that remains unanswered is how exactly NF works to reduce pain intensity. Although a given NF protocol usually seeks to alter brain activity in a specific way, as noted previously, researchers do not always include a manipulation check to determine if (and how much) brain activity changed as intended. Moreover, when such checks are performed, the findings indicate that even if the treatment protocol was effective for reducing pain, it was not always effective for changing brain activity as originally intended (Rogala et al., 2016; Omejc et al., 2018). In fact, in many studies the changes in pain intensity or frequency occurred irrespective of whether the targeted brain activity modulation occurred (Siniatchkin et al., 2000; Jensen et al., 2013b; Emmert et al., 2014; Rance et al., 2014b; Guan et al., 2015). It remains possible that much, if not all, of the beneficial effects of many NF protocols are due to their non-specific effects (e.g., effects on patient outcome expectancies, or effects on mechanisms that may be shared across different NF protocols, such as perceived self-efficacy), as argued by Thibault and colleagues (Thibault et al., 2017).

Mechanism research is needed to address the specificity of NF treatment. For example, participants in a clinical trial could be randomly assigned to one of three conditions: (1) a condition targeting an increase in a specific bandwidth power; (2) a condition that seeks to normalize power across all bandwidths, based on the results of a pre-treatment qEEG assessment; or (3) a control condition (e.g., sham EEG or a protocol that seeks to decrease α power). qEEG could be assessed before and after treatment sessions, during one or more of the treatment sessions and before and after treatment. A finding that participants in one or the other of the experimental conditions report larger improvements in pain than participants in the control condition could be used as evidence for the potential specificity of NF's effects. Even more importantly, additional evidence for treatment specificity could come from mediation analyses to determine the extent to which pre- to post-treatment changes in the power of one or more bandwidths or the ability of an individual to alter bandwidth power during a treatment session mediates the beneficial effects of the experimental conditions relative to the control condition. One example of such a mediation analyses performed in the context of an exploratory study was recently published by Prinsloo and colleagues (Prinsloo et al., 2019). They found that changes in the current source density in two of the targeted Brodmann's areas (the ventral and the dorsal parts of the ACC) completely mediated the reduction in pain intensity achieved with LORETA Z-score NF in patients with cancer undergoing radiation therapy (Prinsloo et al., 2019). This finding provides preliminary support for the specific effects of the NF protocol examined, and points to the activity in the ACC as a potential mechanism for NF interventions that should be examined in future NF studies.

### Study Quality

The fourth and final aim of this review was to assess the quality of the studies included. The results of the quality analysis were mixed. On one hand, all but two of the studies were rated as either “strong” or “moderate” with respect to study design. It is important to note that “strong” study quality is a rating assigned to RCTs or controlled clinical trials, whereas the “moderate” study quality is a rating assigned to studies with a pre-post design, with either just one cohort or with a control group, or case-control studies. It is also important to note that only three studies included in the review were RCTs. Also, more than half of the studies used reliable and valid measures to assess outcomes and were rated as “strong” with respect to withdrawals and drop-outs. On the other hand, most of the studies reviewed had relatively small sample sizes and were pilot studies.

In order to maximize the quality of future clinical trials in this area, so that future systematic reviews could draw more definitive conclusions regarding the efficacy of NF for pain management, researchers should ponder several important study quality considerations. First, future studies would benefit from more robust experimental designs and a more homogeneous and clearer reporting of the protocols and outcomes of the study. In order to achieve this, consensus recommendations on the reporting and experimental design of clinical and cognitive-behavioral neurofeedback studies was recently published (Ros et al., 2020). These recommendations could serve as a framework for the design, conduct, and reporting NF studies.

Second, it is necessary for future studies to estimate sample sizes a priori, ensuring they are adequate for the planned statistical analyses. To be on the safe side, given that pilot studies often over-estimate effect sizes, researchers should seriously consider exceeding the estimated sample size. This would also help to ensure that the samples are large enough to allow for drop-outs or potential missing data.

Third, less than a third of the studies included in this review conducted follow-up assessments. This issue does not allow us to determine if the gains in the studies that did find a reduction in pain intensity or pain frequency at posttreatment were maintained for any period of time after treatment. For NF to be recommended, future studies should consistently report NF effects after treatment and in successive follow-ups.

Fourth, with rare exceptions, most of the studies included in this review used adult samples. As chronic pain is also highly prevalent in children and adolescents (Huguet and Miró, 2008), it would be essential to include these segments of the population in future studies in order to ascertain NF's efficacy in youths.

Fifth, most of the studies did not report several confounding factors, such as medication intake and duration of the problem. Thus, we were not able to take into account the moderating effect of these factors in the effects of NF on pain.

Finally, detailed information about the studies was often lacking. For example, the interventions were often not described in enough detail to allow for replicability. Moreover, detail was sometimes lacking in the description of the outcomes (e.g., reporting decrease or increase percentages only, rather than specific baseline and post-treatment numbers in addition to percentages). In addition to the fact that some studies did not report brain activity information, those which did reported a large variety of variables; it appears that there are no standards yet for reporting basic brain activity information. All of these limitations prevented us from encapsulating and drawing firm conclusions on the efficacy of NF to modulate pain.

### Future Studies

In order to improve on the quality and utility of clinical trials, future studies should seek to identify the protocols that work best for each pain condition, the number of sessions needed to see improvements, the brain mechanisms involved, and how long the improvements are maintained after treatment (Van Boxtel and Gruzelier, 2014), both in youths and adults. However, it is possible that determining fixed protocols for each condition might not be the best path, and instead, tailored protocols for each individual might be better to improve the efficacy of NF studies (Rogala et al., 2016). Also, and in light of some researchers questioning the benefits of NF over and above placebo (Thibault et al., 2017), future studies should consider including a placebo condition. In our review, only three studies controlled for possible placebo effects; for example, by targeting a brain region or a frequency band assumed to be unrelated to pain processing.

### Limitations

There are a number of limitations to this review that should be acknowledged. Because we sought to summarize the evidence of NF used to modulate any type of pain, inclusion criteria were broad. As a result, the included studies were highly heterogeneous, so that we were not able to conduct a meta-analysis. Another limitation is that our data search was limited to studies published in either English or Spanish. It is possible that we overlooked some additional relevant contributions to the field published in journals written in additional languages.

### Summary and Conclusions

This review provides positive preliminary evidence of NF as a potential treatment for chronic pain. However, higher quality studies using similar procedures and outcome measures are still needed to: (1) determine the extent to which promising preliminary studies replicate in order to determine if NF is effective, (2) elucidate the mechanisms of NF treatments on pain, and (3) determine the best NF approach(es) for individuals with chronic pain.

## Author Contributions

RR conceptualized the study, created the research strategy, selected the studies, and extracted the data. RV contributed to the previous tasks and independently selected the studies. JM and MJ supervised all the steps of the process. All authors participated in the interpretation of the data, drafted the work and revised it critically for important intellectual content, and provided approval for publication of the content.

## Conflict of Interest

The authors declare that the research was conducted in the absence of any commercial or financial relationships that could be construed as a potential conflict of interest.
